# Intraprocedural Hemodynamics During Concomitant Rheocarna Therapy and Maintenance Hemodialysis: Effects of Dialyzer Membrane Type and Circuit Sequence

**DOI:** 10.1002/jca.70184

**Published:** 2026-10-04

**Authors:** Yusuke Ishizuka, Genshin Sato, Shizuka Ishigaki, Ryo Enoki, Kako Kajiwara, Akihiko Nakasato, Sosuke Kinomura, Saori Kin, Yuji Noguchi, Daisuke Yoshizawa, Koichi Kikuchi, Michiaki Abe, Koki Kumagai, Tetsuhiro Tanaka, Koji Okamoto

**Affiliations:** ^1^ Department of Nephrology, Graduate School of Medicine Tohoku University Sendai Japan; ^2^ Hemodialysis Unit Minamisanriku Hospital Minamisanriku Japan

**Keywords:** AN69 membrane, chronic limb‐threatening ischemia, hemodialysis, LDL apheresis

## Abstract

Chronic limb‐threatening ischemia (CLTI), a severe manifestation of peripheral artery disease, is particularly prevalent among patients receiving maintenance hemodialysis (HD) and is associated with poor limb and survival outcomes. Low‐density lipoprotein (LDL) apheresis, including Rheocarna, is used as adjunctive therapy for refractory CLTI; however, intraprocedural hypotension remains a major limitation, especially when combined with HD. We examined the effects of dialyzer membrane type and extracorporeal circuit sequence on hemodynamic stability during concomitant therapy. This case series comprised two patients with CLTI receiving maintenance HD. Each patient underwent four treatment sessions representing the four configurations defined by two dialyzer membranes (polysulfone [PS] and AN69) and two circuit sequences (Rheocarna → dialyzer vs. dialyzer → Rheocarna). Hemodynamic parameters, including systolic blood pressure, relative blood volume change (ΔBV), and peripheral microvascular perfusion assessed by laser Doppler flowmetry (ΔLDF), were assessed during each treatment session. In both cases, the Rheocarna → AN69 configuration showed the smallest early decrease in systolic blood pressure and the most favorable early ΔBV pattern among the four sessions. Toe ΔLDF was preserved or increased with this configuration. The other three configurations were associated with larger early decreases in systolic blood pressure in one or both patients, including a decrease to below 90 mmHg in Case B. These findings suggest that dialyzer membrane type and circuit sequence may influence intraprocedural hemodynamic stability during concomitant Rheocarna therapy and HD. Among the tested configurations, Rheocarna → AN69 showed better hemodynamic tolerance and preservation of peripheral perfusion. These hypothesis‐generating findings warrant further evaluation in larger prospective studies.

## 1 Introduction

Chronic limb‐threatening ischemia (CLTI) is the most severe manifestation of peripheral artery disease (PAD) and is characterized by ischemic rest pain, ulceration, or gangrene. Patients receiving maintenance hemodialysis (HD) have a particularly high risk of CLTI because kidney failure contributes to medial arterial calcification, diffuse below‐the‐knee arterial disease, and accelerated atherosclerosis [1, 2, 3]. Compared with patients not receiving HD, patients receiving HD have more severe medial and intimal calcification of the lower‐extremity arteries, particularly below the knee, as well as more extensive atherosclerotic lesions [4].

PAD has been reported in 17%–48% of patients receiving HD; in Japan, CLTI has been reported in 8.6% of patients receiving maintenance HD [5, 6]. In this population, CLTI is associated with infection, intradialytic hypotension, lower limb amputation, impaired quality of life, and increased mortality [2, 7, 8, 9]. Preventing progression from PAD to CLTI is therefore critical for avoiding amputation and improving overall survival.

Low‐density lipoprotein (LDL) apheresis is an extracorporeal therapy that uses negatively charged adsorption columns to selectively remove LDL cholesterol and, depending on the device, fibrinogen, potentially improving hemorheology and peripheral circulation. Indications under the Japanese National Health Insurance system are device specific: Liposorber, which is approved in the United States for LDL apheresis in selected patients with familial hypercholesterolemia, including certain patients with documented coronary artery disease or PAD, is indicated in Japan for patients with Fontaine Stage II or higher disease who meet lipid criteria despite medical therapy (total cholesterol ≥ 220 mg/dL or LDL cholesterol ≥ 140 mg/dL) and have treatment‐resistant occlusive arterial disease. In contrast, Rheocarna is a direct whole‐blood hemoperfusion that does not require plasma separation and is reserved for patients with Fontaine Stage IV disease, particularly patients with nonhealing ulcers for whom revascularization is infeasible or remains inadequate [10, 11].

In patients undergoing HD, the requirement for bed rest (typically 4–5 h per session, three times per week) is already substantial. In addition, Liposorber‐based LDL apheresis requires 2–3 h per session and is generally performed 1–2 times per week, with Japanese National Health Insurance coverage limited to 10 sessions over 3 months, whereas Rheocarna therapy is generally performed for approximately 2 h per session, three times per week, with coverage limited to 24 sessions over 3 months. Scheduling these therapies on dialysis or non‐dialysis days can substantially affect quality of life (QOL) and the maintenance of activities of daily living (ADL). Concurrent treatment therefore offers clear practical advantages.

Treatment‐related hypotension may nevertheless limit concomitant therapy. During LDL apheresis, hypotension may occur within the first hour of treatment concurrently with rising bradykinin concentrations [12, 13]. Intradialytic hypotension is also associated with increased mortality in patients receiving HD [2, 14, 15, 16].

The AN69 membrane, a copolymer of acrylonitrile and sodium methallylsulfonate, is highly permeable and has selective adsorptive properties. A recent review suggested that AN69 membranes may improve nutritional status, reduce inflammation, and decrease amino acid loss in patients undergoing HD [17]. Among patients receiving HD who have CLTI, AN69 membranes were associated with improved peripheral perfusion as assessed by skin perfusion pressure and thermography, as well as with improved clinical symptoms [18]. In addition, the AN69 membrane provides dialysis efficiency comparable to that of PS membranes for the removal of small‐molecular‐weight solutes and positively charged substances but shows significantly lower β2‐microglobulin removal [18, 19]. In a crossover study of 28 older patients undergoing maintenance HD, saline administration for hypotensive episodes was less frequent with AN69 membranes than with polysulfone membranes, suggesting improved intradialytic hemodynamic stability [20]. Although direct evidence in the setting of concomitant LDL apheresis and HD remains limited, these findings suggest that AN69 membranes may improve hemodynamic tolerance during concomitant LDL apheresis and HD.

Dialyzer membrane type and circuit sequence may both influence hemodynamic stability during concomitant Rheocarna and HD. To our knowledge, concurrent Rheocarna therapy and HD have been reported in only three patients, and the effects of circuit sequence and dialyzer membrane type on intraprocedural hemodynamics have not been systematically evaluated [12, 21]. We therefore report a two‐patient case series that compares hemodynamic responses across four combinations of these factors.

## 2 Materials and Methods

All procedures were performed under the Japanese National Health Insurance system during conventional maintenance hemodialysis (HD), with 4‐h sessions performed three times weekly. The target processed blood volume for Rheocarna was 6 L per session [22, 23]. Before combination therapy, each patient had tolerated separate sessions of Rheocarna therapy and HD with the AN69 dialyzer without membrane‐related hypersensitivity reactions or other adverse events. Each patient was evaluated under four treatment conditions in separate sessions, defined by two dialyzer membranes (PS and AN69) and two circuit sequences (Rheocarna → dialyzer and dialyzer → Rheocarna).

As shown in Figures 1 and 2, HD and LDL adsorption were performed concurrently at a blood flow rate (QB) of 100 mL/min for the first hour in all treatment conditions. In the Rheocarna → dialyzer configuration, blood passed through the Rheocarna adsorption column before the dialyzer; the reverse sequence was used in the dialyzer → Rheocarna configuration. After the 1‐h LDL adsorption session, blood remaining in the LDL adsorption segment of the extracorporeal circuit was returned to the patient, after which HD alone continued at a QB of 250 mL/min. The prescribed machine ultrafiltration rate remained constant throughout each 4‐h HD session, although total ultrafiltration volume differed among sessions.

**FIGURE 1:**
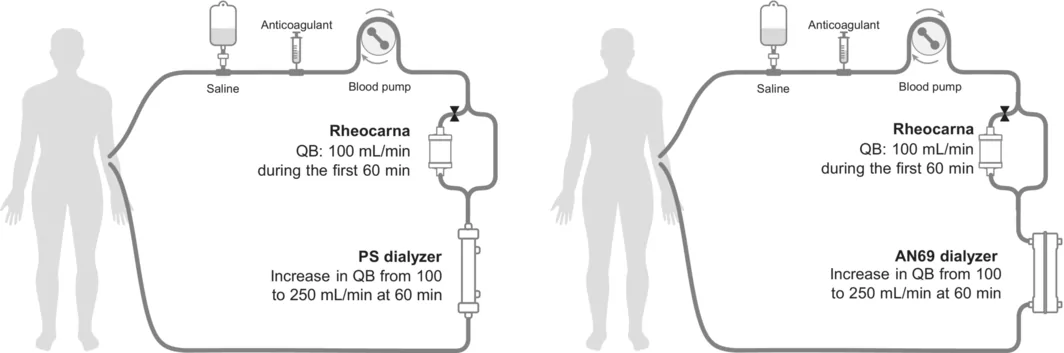
Schematic of the Rheocarna → dialyzer circuit configuration during concomitant Rheocarna therapy and HD. (a) Rheocarna → PS. (b) Rheocarna → AN69. For the first hour, HD and LDL adsorption were performed concurrently at a QB of 100 mL/min. Blood passed first through the Rheocarna column and then through the dialyzer. After the 1‐h adsorption session, residual blood in the LDL adsorption segment was returned to the patient, and HD then continued alone at a QB of 250 mL/min.

**FIGURE 2:**
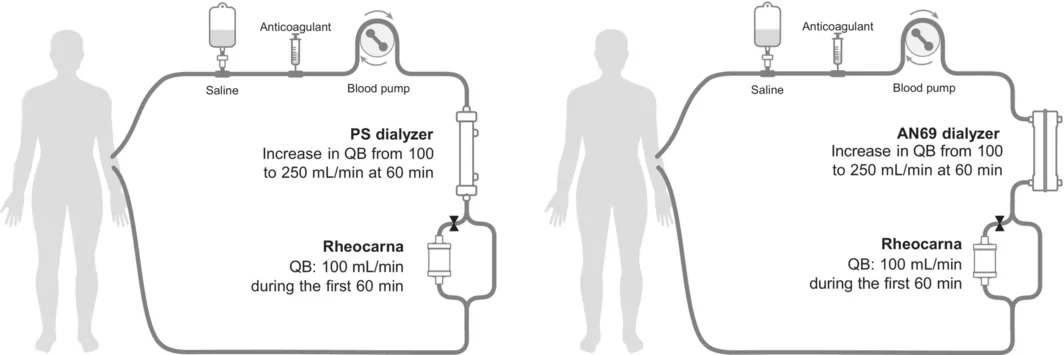
Schematic of the dialyzer → Rheocarna circuit configuration during concomitant Rheocarna therapy and HD. (a) PS → Rheocarna. (b) AN69 → Rheocarna. For the first hour, HD and LDL adsorption were performed concurrently at a QB of 100 mL/min. Blood passed first through the dialyzer and then through the Rheocarna column. After the 1‐h adsorption session, residual blood in the LDL adsorption segment was returned to the patient, and HD then continued alone at a QB of 250 mL/min.

The PS hollow‐fiber dialyzer APS‐25EA (Asahi Kasei Medical, Japan) served as the control dialyzer. For the AN69 condition, we used an H12‐4000 hemodialyzer (Baxter, Japan), a stacked‐type dialyzer equipped with a hydrophilic AN69 membrane composed of acrylonitrile and sodium methallylsulfonate with adsorptive properties. Rheocarna (Kaneka Medix, Osaka, Japan) is an adsorption‐based blood purification device that selectively removes low‐density lipoprotein cholesterol and fibrinogen. Neither patient received an angiotensin‐converting enzyme inhibitor during the evaluated sessions. Unfractionated heparin was administered for anticoagulation as an initial bolus of 1000 units followed by a continuous infusion of 1000 units/h. If hypotension occurred, glycerol, etilefrine, and saline were administered as needed. Intradialytic hypotension was defined as a systolic blood pressure of < 90 mmHg, or a decrease in systolic blood pressure of ≥ 20 mmHg associated with symptoms such as nausea or headache [24].

We assessed the effects of dialyzer membrane type and circuit sequence on intraprocedural hypotension. In addition to intermittent blood pressure measurements at 30‐min intervals, relative blood volume change (ΔBV) and peripheral skin microvascular perfusion were assessed as indicators of systemic and peripheral hemodynamic responses [25]. ΔBV was monitored every 20 s using a BVplus monitor integrated into the dialysis machine (DCS‐200Si; Nikkiso, Tokyo, Japan). The system optically estimates hematocrit from near‐infrared light reflected by blood in the extracorporeal circuit and calculates ΔBV from the resulting hematocrit changes. The device output was re‐referenced to 0% at the start of concomitant Rheocarna therapy and HD; negative values indicate a decrease in ΔBV from baseline [26]. Measurements were obtained every 20 s, and the corresponding figures present 5‐min averages. Peripheral skin microvascular perfusion was assessed using laser Doppler flowmetry at the plantar aspect of the great toe [27]. LDF measurements were obtained every minute. ΔLDF was expressed as the percentage change from baseline at treatment initiation; the corresponding figure presents 5‐min averages.

## 3 Case Presentations

### 3.1 Case A

Case A was a 64‐year‐old man with type 2 diabetes mellitus and kidney failure who had been receiving maintenance HD since age 46 for diabetic kidney disease. He had previously undergone stent‐graft placement for arteriovenous graft occlusion, and aspirin was continued to prevent recurrent graft occlusion. He received a calcium channel blocker only on non‐dialysis days and an angiotensin II receptor blocker daily at noon. He received neither an angiotensin‐converting enzyme inhibitor nor lipid‐lowering therapy.

At age 62, the patient developed osteomyelitis of the distal phalanx of the left middle finger. At age 63, he developed a left fifth‐toe ulcer in the setting of Fontaine Stage IV CLTI. The ulcer was complicated by osteomyelitis diagnosed based on radiographic findings. He also experienced occasional rest pain in the affected foot. Initial incision and drainage did not improve the lesion. He subsequently underwent negative‐pressure wound therapy, debridement, and stump revision. He continued daily cleansing of the affected limb and routine foot care as instructed. Despite these interventions, the ulcer progressively worsened.

The ankle–brachial index was 0.81 on the right and 0.72 on the left, and the left dorsalis pedis pulse was not palpable. Angiography showed diffuse bilateral stenoses from the superficial femoral to the popliteal arteries, with no distal runoff below the popliteal arteries. Revascularization was therefore considered infeasible on technical grounds. Rheocarna therapy was initiated 2 months after the initial incision and drainage because the ulcer continued to worsen despite surgical treatment and wound care. Baseline clinical characteristics, dialysis conditions, and laboratory values at the initiation of concomitant Rheocarna therapy and HD are summarized in Table 1.

**TABLE 1:** Baseline characteristics and laboratory data of the two patients.

Characteristics	Case A	Case B	Unit
Patient characteristics			
Age at CLTI diagnosis	63	53	years
Age at Rheocarna initiation	64	62	year
Dialysis duration	18	17	year
Sex	Male	Male	
Dry weight	69.5	67.5	kg
Vascular access	AV graft	AV fistula	
Medications during HD			
Darbepoetin alfa	None	30	μg/week
Etelcalcetide	30	5	mg/week
Etilefrine	None	2	mg/h
Glycerol	None	40	mL/h
Levocarnitine	1000	1000	mg/week
Maxacalcitol	None	2.5	μg/week
Hematologic parameters			
White blood cell count	6.5	9.8	×10^3^/μL
Red blood cell count	2.9	4.1	×10^6^/μL
Hemoglobin	10.3	11.6	g/dL
Hematocrit	32.6	35.0	%
Platelet count	132	231	×10^3^/μL
Biochemical parameters			
Blood urea nitrogen	14.1	11.6	mg/dL
Creatinine	10.4	3.0	mg/dL
Aspartate aminotransferase	23	11	U/L
Alanine aminotransferase	16	5	U/L
Alkaline phosphatase	138	51	U/L
γ‐Glutamyl transferase	12	12	U/L
Lactate dehydrogenase	318	190	U/L
Uric acid	2.1	1.7	mg/dL
Total protein	5.6	6.5	g/dL
Albumin	3.0	3.3	g/dL
Total cholesterol	136	115	mg/dL
High‐density lipoprotein cholesterol	35	58	mg/dL
Low‐density lipoprotein cholesterol	85	48	mg/dL
Triglycerides	93	74	mg/dL
Sodium	136	135	mEq/L
Potassium	3.3	3.2	mEq/L
Chloride	101	100	mEq/L
Total calcium	8.1	7.9	mg/dL
Phosphorus	1.4	1.8	mg/dL
Magnesium	2.2	2.0	mg/dL
C‐reactive protein	0.68	0.24	mg/dL
Glycated albumin	19.1	15.9	%
*β*2‐Microglobulin	30.4	30.4	mg/L
Intact parathyroid hormone	47	247	pg/mL

*Note:* Laboratory values were obtained before dialysis on the day of the first concomitant Rheocarna–HD session.

Abbreviations: AV, arteriovenous; CLTI, chronic limb‐threatening ischemia; HD, hemodialysis.

After 24 sessions of Rheocarna therapy, the ankle–brachial index increased to 0.85 on the right and 0.84 on the left. Episodes of rest pain became less frequent, and the previously nonpalpable left dorsalis pedis pulse was weakly palpable. No further CLTI treatment was required during the subsequent year. The clinical course of Case A is summarized in Figure 3a.

**FIGURE 3:**
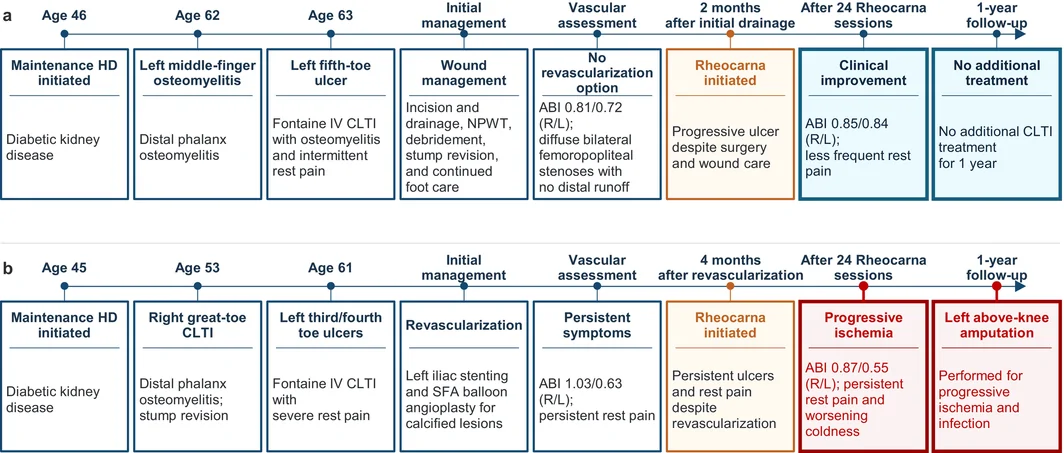
Clinical timelines of Cases A and B. The timelines show the major clinical events and outcomes. (a) In Case A, a left fifth‐toe ulcer progressed despite wound care, and revascularization was deemed infeasible. Rheocarna therapy was initiated 2 months after drainage; after 24 sessions, ABI and rest pain improved, and no additional CLTI treatment was required during the 1‐year follow‐up. (b) In Case B, ulcers and rest pain persisted despite revascularization. Rheocarna therapy was initiated 4 months later, but ischemia and infection progressed and led to left above‐knee amputation. Orange boxes indicate Rheocarna initiation, whereas red boxes indicate disease progression and amputation. The timelines are not drawn to scale.

### 3.2 Case B

Case B was a 62‐year‐old man with hypertension, type 2 diabetes mellitus, and a history of myocardial infarction. Maintenance HD had been initiated at age 45 for diabetic kidney disease. Aspirin and pitavastatin were continued, whereas he was not receiving an angiotensin‐converting enzyme inhibitor. During maintenance HD, he routinely required glycerol and continuous etilefrine infusion at 2 mg/h for hemodynamic support. Transthoracic echocardiography revealed severe mitral regurgitation with relatively preserved left ventricular systolic and diastolic function.

At age 53, he developed CLTI with osteomyelitis of the distal phalanx of the right great toe and subsequently underwent right great‐toe stump revision. At age 61, worsening discoloration and cyanosis of the left third and fourth toes were followed by nonhealing ulcers. The lesions were classified as Fontaine Stage IV and were accompanied by severe rest pain.

Angiography revealed a heavily calcified stenosis of the left common iliac artery and severe stenoses of both the left superficial femoral and profunda femoris arteries. The peroneal artery was the only patent infrapopliteal artery, with patency extending only to the ankle level. Because of severe rest pain and progressive CLTI, stent placement in the left common iliac artery and plain old balloon angioplasty of the left superficial femoral artery were performed.

One month after revascularization, the patient continued to receive routine foot care. However, the ankle–brachial index remained low (1.03 on the right and 0.63 on the left), and rest pain persisted. Because the ischemic symptoms and ulcers remained refractory, adjunctive Rheocarna therapy was initiated 4 months after revascularization. Baseline clinical characteristics, dialysis conditions, and laboratory values at the initiation of concomitant Rheocarna therapy and HD are summarized in Table 1.

Despite Rheocarna therapy, the ankle–brachial index was 0.87 on the right and 0.55 on the left, and rest pain persisted. Coldness of the left lower limb gradually worsened, and the associated infection became increasingly difficult to control. Consequently, the patient underwent left above‐knee amputation approximately 1 year after revascularization. The clinical course in Case B is summarized in Figure 3b.

## 4 Results

### 4.1 Case A

In both dialyzer → Rheocarna configurations (PS → Rheocarna and AN69 → Rheocarna), each tested at an ultrafiltration rate of 0.59 L/h (8.4 mL/kg/h), systolic blood pressure fell from approximately 150 mmHg to 100 mmHg within 30 min. In both settings, systolic blood pressure gradually recovered to approximately 120 mmHg over the next 30 min without vasopressor support and returned to approximately 150 mmHg after reinfusion of the blood remaining in the Rheocarna circuit. The Rheocarna → PS configuration showed a similar pattern, despite a lower ultrafiltration rate of 0.47 L/h (6.7 mL/kg/h). By contrast, under the Rheocarna → AN69 configuration, despite a higher ultrafiltration rate of 0.71 L/h (10.2 mL/kg/h), systolic blood pressure decreased only from approximately 150 to 120 mmHg at 30 min, after which it remained stable at approximately 150 mmHg.

Toe ΔLDF increased in all conditions, and the Rheocarna → AN69 configuration showed the greatest and most sustained increase (Figure 4a). ΔBV decreased progressively over time under all conditions (Figure 5). After Rheocarna was discontinued at 1 h and the remaining blood was reinfused, ΔBV fell markedly with the PS configuration, whereas the decline appeared attenuated with AN69.

**FIGURE 4:**
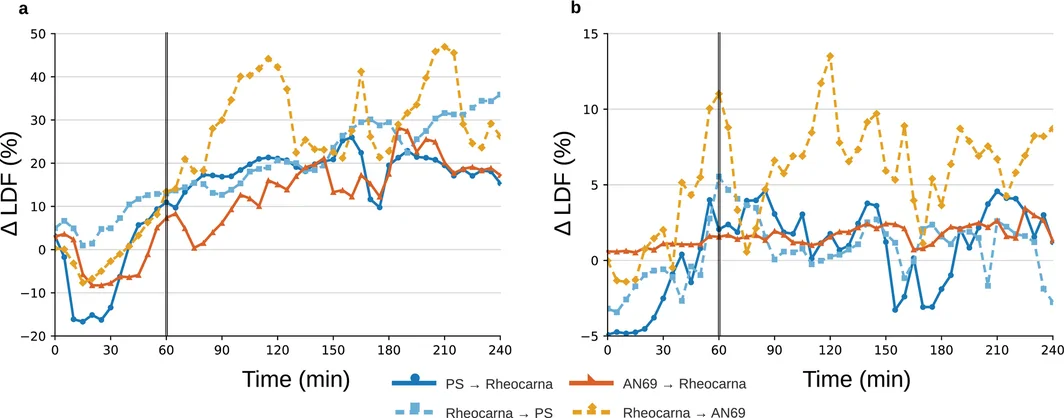
Temporal changes in toe peripheral microvascular perfusion during concomitant Rheocarna therapy and HD under four treatment configurations. (a) Case A. (b) Case B. ΔLDF denotes the percentage change in laser Doppler flux from the value at the start of concomitant treatment. LDF was recorded at 1‐min intervals, and means were plotted for consecutive 5‐min intervals. The four configurations were PS → Rheocarna, AN69 → Rheocarna, Rheocarna → PS, and Rheocarna → AN69. At 60 min, the double vertical line indicates completion of the Rheocarna phase, blood return, and the increase in QB from 100 to 250 mL/min.

**FIGURE 5:**
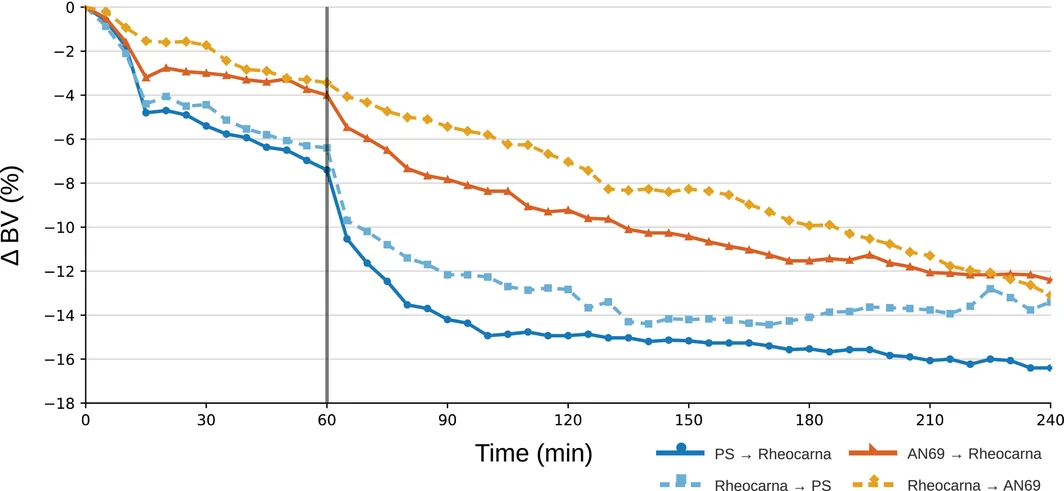
Temporal changes in ΔBV of Case A during concomitant Rheocarna therapy and HD under four treatment configurations. ΔBV was continuously monitored and re‐referenced to 0% at the start of concomitant treatment. Values are presented at 5‐min intervals. At 60 min, the double vertical line marks completion of Rheocarna therapy, blood return, and an increase in QB from 100 to 250 mL/min.

### 4.2 Case B

During maintenance HD, the patient routinely required glycerol and continuous etilefrine infusion (2 mg/h). Under the PS → Rheocarna, AN69 → Rheocarna, and Rheocarna → PS configurations, systolic blood pressure decreased by more than 30 mmHg within 30 min, from 120 to 140 mmHg to below 90 mmHg, despite a total ultrafiltration volume of 3.4 L and an ultrafiltration rate of 12.6 mL/kg/h. By contrast, under the Rheocarna → AN69 configuration, despite a slightly greater total ultrafiltration volume of 3.5 L and an ultrafiltration rate of 12.9 mL/kg/h, systolic blood pressure decreased only from approximately 140–120 mmHg.

Across all four conditions, toe ΔLDF was relatively preserved; the Rheocarna → AN69 configuration showed the greatest and most sustained increase (Figure 4b). ΔBV declined progressively over time across all four conditions (Figure 6). Although the patterns were broadly similar across conditions, the late‐phase decline was greatest under the PS → Rheocarna configuration, whereas the early decline was smallest under the Rheocarna → AN69 configuration.

**FIGURE 6:**
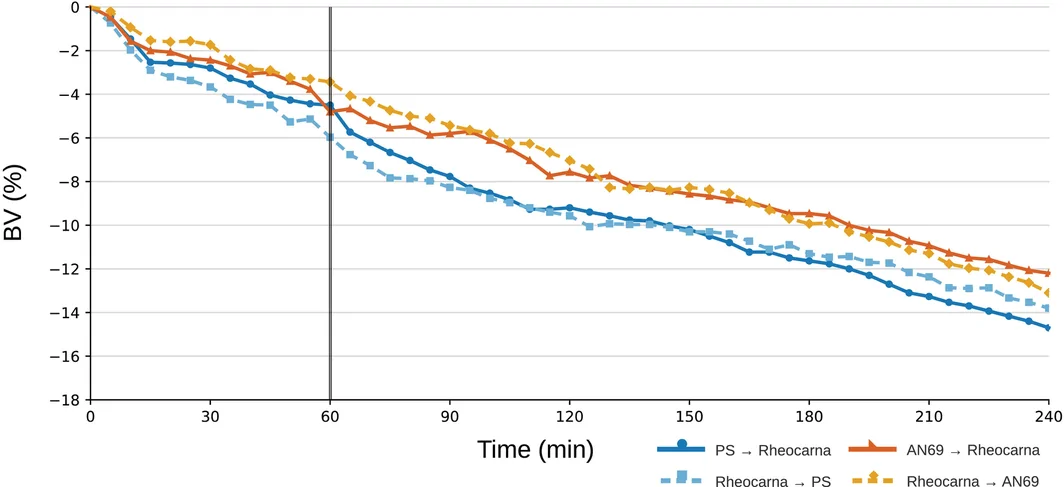
Temporal changes in ΔBV of Case B during concomitant Rheocarna therapy and HD under four treatment configurations. ΔBV was continuously monitored and re‐referenced to 0% at the start of concomitant treatment. Values are presented at 5‐min intervals. At 60 min, the double vertical line marks completion of Rheocarna therapy, blood return, and an increase in QB from 100 to 250 mL/min. Abbreviations: ABI, ankle‐brachial index; AN69, acrylonitrile–sodium methallylsulfonate copolymer membrane; ΔBV, relative blood volume change; CLTI, chronic limb‐threatening ischemia; ΔLDF, percentage change in laser Doppler flux from baseline; NPWT, negative‐pressure wound therapy; HD, hemodialysis; PS, polysulfone membrane; QB, blood flow rate; R/L, right/left; SFA, superficial femoral artery.

### 4.3 Overall Findings

In both cases, the Rheocarna → AN69 configuration was associated with milder hypotension (AN69: 25.5 ± 6.8 mmHg; PS: 37.3 ± 2.1 mmHg) and a smaller ΔBV decrease than the other treatment configurations. In Case A, this configuration showed a smaller early decline in systolic blood pressure and a late increase in ΔLDF despite a gradual decrease in ΔBV. In Case B, it was also associated with a smaller decrease in systolic blood pressure than the other three configurations, while ΔLDF remained relatively preserved.

## 5 Discussion

To our knowledge, this is the first case series to evaluate intraprocedural hemodynamics in patients with CLTI during concomitant Rheocarna therapy and maintenance HD in relation to both dialyzer membrane type and circuit sequence. Among the four configurations tested, the Rheocarna → AN69 configuration showed the smallest early declines in systolic blood pressure and ΔBV and better preservation of peripheral microvascular perfusion. In Case A, this configuration was associated with a smaller early decline in systolic blood pressure despite a higher ultrafiltration rate, followed by a late increase in ΔLDF despite a gradual reduction in ΔBV. In Case B, it was the only configuration in which systolic blood pressure remained above 90 mmHg during the first 30 min, while ΔLDF remained relatively preserved. Although descriptive, these observations suggest that both dialyzer membrane selection and circuit sequence may influence intraprocedural hemodynamic responses during concomitant therapy.

These findings are clinically relevant because patients undergoing HD with CLTI commonly have diffuse, multilevel, predominantly below‐the‐knee arterial disease, severe calcification, impaired distal runoff, endothelial dysfunction, and microvascular abnormalities. Collectively, these features increase the need for adjunctive therapies that may improve tissue perfusion while preserving hemodynamic stability [4, 28, 29]. LDL apheresis can reduce LDL cholesterol, fibrinogen, and coagulation factors, with potential improvement in peripheral microcirculation. Rheocarna may provide related benefits through direct adsorption of LDL‐C and fibrinogen and can be incorporated more readily into an HD circuit because it does not require plasma separation [11]. As fibrinogen contributes to plasma viscosity and stabilizes erythrocyte aggregation even under microcapillary flow conditions, its removal may further improve hemorheology and potentially support microcirculatory flow [30, 31]. In the TURNIP registry, 87.8% of treated patients were receiving dialysis, and Rheocarna therapy was associated with reductions in LDL‐C, fibrinogen, and C‐reactive protein levels and improvements in ABI and SPP. At 1 year, the wound healing and limb salvage rates were 60.7% and 83.4%, respectively [11]. However, 33.0% of patients experienced a decrease in systolic blood pressure of at least 30 mmHg, predominantly during the first hour, and hypotension contributed to treatment discontinuation in some patients. Early hemodynamic tolerance and extracorporeal blood flow management are therefore important considerations in procedural optimization.

Several interacting mechanisms may account for the relatively favorable hemodynamic profile of the Rheocarna → AN69 configuration. First, circuit sequence may affect pressure distribution within the circuit, the composition of returned blood, and systemic exposure to vasoactive mediators. Within a shared circuit, the sequence determines whether blood exiting Rheocarna passes through the dialyzer before returning to the systemic circulation. Accordingly, in the Rheocarna → dialyzer configuration, vasoactive mediators may undergo membrane‐dependent adsorption or transport before the blood returns to the systemic circulation. Because mediator concentrations were not measured upstream and downstream of each device, this interpretation remains hypothetical.

Second, early hemodynamic changes may involve activation of the contact system by negatively charged dextran sulfate immobilized within the Rheocarna adsorbent, leading to factor XII activation, kallikrein formation, and subsequent bradykinin release. Bradykinin is a strongly basic vasoactive nonapeptide with a molecular weight of 1060.2 Da and a reported isoelectric point of approximately 12.0; therefore, it is expected to carry a net positive charge at physiological pH [32, 33, 34]. Its small molecular size and charge render membrane transport and electrostatic interactions with negatively charged extracorporeal surfaces plausible; however, its net handling cannot be inferred from these properties alone. Bradykinin is rapidly degraded, principally by angiotensin‐converting enzyme (ACE) and aminopeptidase P; however, excessive accumulation can cause vasodilation, increased vascular permeability, pain, and hypotension [35, 36]. Accordingly, ACE inhibitors are contraindicated during Rheocarna therapy, and a recent case series showed that intraprocedural blood pressure reduction may coincide with increased circulating bradykinin concentrations.

Bradykinin may have opposing effects on lower‐extremity arterial disease. Moderate B2‐receptor‐mediated vasodilation may theoretically improve regional microvascular flow, whereas excessive vasodilation may lower systemic blood pressure, reduce effective perfusion pressure, increase vascular permeability, induce pain, and transiently impair tissue oxygenation [37]. During Rheocarna therapy, increases in circulating bradykinin are accompanied by reductions in blood pressure and transcutaneous oxygen tension, although oxygen tension subsequently recovers by the end of the treatment [13]. Thus, although the role of bradykinin in the medium‐ or long‐term therapeutic effects of Rheocarna remains uncertain, transient bradykinin elevation may contribute to acute intraprocedural hypotension and related symptoms.

Third, native AN69 has membrane‐specific properties that may affect the handling of positively charged vasoactive mediators. Native AN69 is not surface‐treated with polyethyleneimine; its hydrophilic, sulfonate‐rich hydrogel matrix contains fixed anionic sites that enable electrostatic and bulk adsorption of positively charged proteins and inflammatory mediators [38]. AN69 membranes have also been associated with reduced inflammation and amino acid loss and with improved peripheral perfusion in patients receiving HD, including those with CLTI [17, 18, 19]. Direct evidence during concomitant Rheocarna therapy and HD remains limited; however, these properties provide a possible mechanistic basis for the relatively favorable hemodynamic profile of the Rheocarna → AN69 configuration.

Consistent with these mechanistic considerations, the relatively favorable profile of Rheocarna → AN69 was not limited to systolic blood pressure but was also reflected in ΔLDF. The preservation or increase in ΔLDF despite a progressive decline in ΔBV suggests that local peripheral perfusion was not determined solely by changes in intravascular volume and may also have depended on vascular tone and local hemorheology [1, 2]. However, laser Doppler flowmetry measures perfusion within a small tissue volume and is sensitive to probe position, temperature, and local conditions. Therefore, these findings should not be interpreted as evidence of improved perfusion throughout the entire limb [39].

From a practical perspective, our cases highlight a potentially useful technical point. Separate HD and Rheocarna sessions are conceptually simpler but prolong total treatment time and increase the procedural burden for both patients and healthcare providers. Concurrent therapy through separate vascular access routes would permit independent control of each circuit but would require two extracorporeal circuits, thereby increasing the extracorporeal blood volume and potential blood loss if circuit clotting occurs. A serial‐parallel circuit incorporating both devices may limit these risks while allowing independent adjustment of QB through the Rheocarna segment. Before combination therapy, both patients underwent separate Rheocarna therapy and HD using an AN69 dialyzer without membrane‐related hypersensitivity reactions or other adverse events, allowing individual tolerability to be confirmed before the two modalities were combined in a shared circuit. In this protocol, both patients subsequently completed concomitant HD and Rheocarna therapy under all four tested conditions by adjusting QB and using an appropriate circuit design, without abandoning either modality. However, the concomitant phase required a reduced QB of 100 mL/min, after which HD alone continued at a higher QB. Although concomitant treatment may reduce total treatment time and procedural burden, prolonged use of a low QB could compromise dialysis efficiency. The dialysis prescription should therefore account for the duration of the 100‐mL/min concomitant phase, the subsequent increase in QB after completion of Rheocarna therapy, and the delivered dialysis dose to balance hemodynamic tolerance with adequate dialysis delivery.

Some limitations should be considered when interpreting these findings. First, this two‐patient case series tested each condition only once and did not randomize treatment order; session‐to‐session variability and changes in clinical status over the treatment course may therefore have affected the findings, and within‐patient reproducibility was not established. Second, systolic blood pressure, ΔBV, and ΔLDF provide no direct information on cardiac output, systemic vascular resistance, absolute circulating blood volume, plasma refilling, or global limb perfusion. Third, mechanistic biomarkers, including bradykinin, nitric oxide, prostaglandins, inflammatory cytokines, and contact activation markers, were not measured upstream or downstream of each device. Fourth, all sessions used heparin for anticoagulation; this is relevant because nafamostat mesylate suppresses bradykinin generation [40]. Fifth, other dialysis prescription factors, including dialysate temperature, ultrafiltration profiling, vasopressor use, and delivered dialysis dose, were neither systematically varied nor evaluated and may have influenced treatment tolerance. Finally, these short‐term observations cannot establish whether circuit configuration affects wound healing, limb salvage, or other long‐term clinical outcomes. Despite these limitations, the observations suggest that dialyzer membrane type and circuit sequence may influence intraprocedural hemodynamic tolerance during concomitant Rheocarna therapy and HD.

## 6 Conclusions

In this case series of two patients, dialyzer membrane type and circuit sequence may influence intraprocedural hemodynamics during concomitant Rheocarna therapy and HD. Among the four configurations, Rheocarna → AN69 showed the most favorable profile, with smaller early decreases in systolic blood pressure and ΔBV and better preservation of peripheral microvascular perfusion. Although larger prospective studies are needed to confirm these findings, modifying the dialysis circuit rather than the treatment schedule may offer a simple, clinically applicable strategy for improving patient care.

## Funding

This manuscript was supported by Japan Society for the Promotion of Science (JSPS) Grants‐in‐Aid for Scientific Research under Grant Number 25K15951 (K.O.).

## Ethics Statement

The requirement for institutional ethics committee approval was waived in accordance with institutional policy for case reports.

## Consent

Written informed consent for publication of the patients' clinical information and accompanying figures was obtained from both patients.

## Conflicts of Interest

The authors declare no conflicts of interest.

## Data Availability

The data supporting the findings of this study are available from the corresponding author upon reasonable request, in accordance with institutional and ethical regulations.
